# The impact of a combined TB/HIV intervention on the incidence of TB infection among adolescents and young adults in the HPTN 071 (PopART) trial communities in Zambia and South Africa

**DOI:** 10.1371/journal.pgph.0001473

**Published:** 2023-07-14

**Authors:** Kwame Shanaube, Ab Schaap, Linda Mureithi, Modupe Amofa-Sekyi, Robynn Paulsen, Maina Cheeba, Bxyn Kangololo, Redwaan Vermaak, Carmen Sisam, Barry Kosloff, Petra de Haas, Sarah Fidler, Maria Ruperez, Richard Hayes, Sian Floyd, Helen Ayles, on behalf of the TREATS Study Team

**Affiliations:** 1 Zambart, Lusaka, Zambia; 2 Department of Infectious Disease Epidemiology, London School of Hygiene and Tropical Medicine, London, United Kingdom; 3 Health Systems Trust, Health Systems Research Unit, Cape Town, South Africa; 4 Clinical Research Department, London School of Hygiene & Tropical Medicine, London, United Kingdom; 5 KNCV Tuberculosis Foundation, The Hague, The Netherlands; 6 Department of Infectious Disease, Faculty of Medicine, Imperial College, London, United Kingdom; Kamuzu University of Health Sciences (KUHeS), MALAWI

## Abstract

**Background:**

HPTN071 (PopART) was a cluster randomized trial conducted in Zambian and South African (SA) communities, between 2013–2018. The PopART intervention (universal HIV-testing and treatment (UTT) combined with population-level TB symptom screening) was implemented in 14 communities. The TREATS study (2017–2021) was conducted to evaluate the impact of the PopART intervention on TB outcomes. We report on the impact of the combined TB/HIV intervention on the incidence of TB infection in a cohort of adolescents and young adults (AYA) aged 15–24 years.

**Methods:**

A random sample of AYA was enrolled between July 2018 and July 2019 in 7 intervention vs 7 standard-of-care communities. We collected questionnaire data on risk factors for TB, and blood for measuring TB infection using QuantiFERON (QFT) Plus. AYA were seen at months 12 and 24 with all procedures repeated. Primary outcome was incidence of TB infection comparing intervention and standard-of-care communities. An incident case was defined as a participant with QFT interferon-gamma response of < 0.2 IU/ml plasma (‘negative’) at baseline and a QFT interferon-gamma response of > = 0.7 IU/ml (‘positive’) at follow up.

**Results:**

We enrolled 4,648 AYA, 2,223 (47.8%) had a negative QFT-plus result at baseline, 1,902 (85.6%) had a follow up blood sample taken at 12 months or 24 months. Among the 1,902 AYA, followed for 2,987 person-years, 213 had incident TB infection giving (7.1 per 100 person-years). TB infection incidence rates were 8.7 per 100 person-years in intervention communities compared to 6.0 per 100 person-years in standard-of-care communities. There was no evidence the intervention reduced the transmission of TB (incidence-rate-ratio of 1.45, 95%CI 0.97–2.15, p = 0.063).

**Conclusion:**

In our trial setting, we found no evidence that UTT combined with TB active case finding reduced the incidence of TB infection at population level. Our data will inform future modelling work to better understand the population level dynamics of HIV and TB.

## Introduction

Worldwide, tuberculosis (TB) is currently the second leading infectious cause of death after COVID-19 [1]. Zambia and South Africa (SA) are among the 30 WHO high TB burden countries (HBCS) that account for 86% of all estimated incident cases of TB disease [1]. In 2021, an estimated 60,000 people were diagnosed with TB in Zambia (estimated incidence rate of 307/100 000 per year), of whom ~50% were coinfected with HIV [1]. In the same year in SA, an estimated 307,000 people were diagnosed with TB (estimated incidence rate of 513/100 000 per year) of whom ~53% were coinfected with HIV [1]. TB is also a leading cause of mortality and morbidity in people living with HIV (PLHIV) in sub-Saharan Africa (SSA) [2].

Globally, TB incidence was falling at about 2% per year, with a cumulative reduction of 11%, between 2015 and 2020. This was only half of the End TB Strategy milestone of a 20% reduction between 2015 and 2020 [1]. Furthermore, the COVID-19 pandemic reversed years of progress made in the fight to end TB. The disruption to routine TB services increased the number of people with undiagnosed TB, who are a major source of ongoing transmission. For instance, in 2020, an estimated 40% of people with TB in low- and middle-income countries (LMICs) were not diagnosed and notified due to COVID-19 [3].

HIV is the leading driver of TB disease in southern and eastern Africa. A universal HIV testing and treatment (UTT) strategy has been proposed as an important component of HIV combination prevention programmes [4, 5]. Four randomized population-based trials done in SSA demonstrated that UTT can rapidly achieve high population-level viral suppression leading to significant reductions in HIV incidence and mortality [6]. UTT facilitates early initiation of antiretroviral therapy (ART) and has an impact on TB epidemiology, particularly in reducing the incidence of TB disease [7]. Therefore, the UTT strategy has the potential to reduce the TB risk at both an individual and a population level [8, 9].

The population-level impact of ART on HIV-TB rates may occur by three predominant means [8]. First, ART can improve the CD4 cell count profile of a population and therefore decrease progression from infection with *M*. *tuberculosis* to TB disease. Second, HIV-infected individuals on ART receive regular follow-up care, providing the opportunity for improved TB case detection and a decrease in the prevalence of untreated TB. Third, widespread early implementation of ART reduces the HIV incidence rate in the general population and eventually the prevalence of HIV infection [6]. Lastly offering TB Preventive Therapy (TPT) to HIV-positive individuals in care, reduces the risk of progression to TB-disease after infection with *M*. *tuberculosis*. Evidence shows that TPT reduces TB incidence, and also mortality in PLHIV up to 37% independent of ART and is a cost-effective intervention [10].

HPTN 071 (PopART) was the largest of the four community-randomised trials that evaluated the impact of a combination prevention package including UTT in reducing new HIV infections [11]. It was conducted in 21 Zambian and SA communities during 2013–2018 [11]. The PopART intervention package was delivered in 14 communities and included universal HIV testing, immediate ART regardless of CD4-count (7 communities) or ART initiation according to national guidelines (7 communities), and population-level TB symptom screening. Although the PopART intervention encompassed TB case finding, the study was primarily aimed at measuring its impact on HIV incidence. The HPTN 071 (PopART) trial reported a reduction in HIV incidence of about 20% in the 14 intervention communities compared with the 7 standard-of-care communities [11].

The TREATS (TB Reduction through Expanded Antiretroviral Treatment and TB Screening) project was carried out to measure whether the PopART intervention was similarly successful in reducing TB prevalence and the incidence of infection with *M*. *tuberculosis* in the same communities [12]. This paper focuses on the incidence of TB infection study which measured whether the PopART intervention reduced the number of people becoming infected with *M*. *tuberculosis*.

Our overall aim was to determine the impact of the combined TB/HIV intervention on the incidence of TB infection in a cohort of adolescents and young adults (AYA aged 15–24 years. We chose to measure incidence of TB infection in AYA as this is the group with high rates of infection [13, 14]. New TB infections in AYA are more likely to reflect community transmission as a result of changes in social mixing patterns. However, TB infection has traditionally been measured in young children in which it is likely to predominantly reflect household transmission [13, 14].

## Methods

### Study design

The TREATS study followed the HPTN 071 (PopART) trial [11] and was conducted during 2018–2021 in 21 peri-urban communities in Zambia (12 communities) and the Western Cape of SA (9 communities), with a total population of approximately 1 million. The trial design of the HPTN 071 (PopART) trial has been described previously [11, 15]. In the HPTN 071 (PopART) trial, the 21 communities were matched into seven triplets based on geographical area and estimated HIV prevalence. The 21 communities were randomly assigned to Arm A (combination prevention intervention with universal ART), Arm B (the prevention intervention with ART provided according to local guidelines (universal since 2016)), or Arm C (standard-of-care) (S1 Fig). The locations of the trial communities have also been described previously [11, 15].

#### The PopART intervention

The combination prevention intervention was delivered in the 14 arm A and B communities. Trained Community HIV-care Providers (CHiPs) delivered services at annual household visits over 4 years in the intervention communities in a door-to door approach. Details of the combination prevention intervention are provided elsewhere [11, 16].

An integral component of the PopART intervention was the implementation of door-to-door active TB case finding activities. The CHiPs actively screened all household members for TB with a TB symptom screen, collected sputum samples from symptomatic individuals within the household, transported the samples to health care facilities for testing and reported back Xpert MTB/RIF Ultra (Cepheid, Sunnyvale, USA) or smear microscopy results. Sputum samples were collected from individuals who had a cough for ≥2 weeks, unintentional weight loss ≥1.5kg in the last month, or current night sweats, and also from those who were living in the same household as a TB patient. Individuals diagnosed with TB were supported with linkage to care at the local health facilities. Data collected by CHiPs on service delivery were used to evaluate intervention coverage.

The HPTN 071 (PopART) trial followed WHO and national guidelines for TB Preventive Therapy (TPT) which were evolving in Zambia and South-Africa during the time of the study [17–19]. These guidelines recommended daily isoniazid for 6 months for the treatment of latent TB among PLHIV and all household contacts of a TB patient. In intervention communities, the UTT strategy encouraged all HIV-positive individuals to link to HIV care. HIV care was strengthened in health facilities which included provision of TPT to HIV-positive individuals after excluding active TB. In control communities TPT was offered to those in HIV care according to national guidelines but no health facility strengthening was put in place. However, in both intervention and control communities, various challenges including policy and management level factors, supply chain factors, health worker perceptions about TPT and limited demand creation activities constrained the scale up of TPT in Zambia [20].

#### TREATS TB infection cohort

The effect of the PopART intervention on population-level TB infection incidence was measured in the TREATS TB infection cohort that included approximately 300 randomly sampled AYA aged 15–24 years per community who were followed up for 2 years. The infection cohort was enrolled in 14 HPTN 071 (PopART) communities (seven arm A and seven arm C) in Zambia and SA. This restriction to arm A (and not including arm B) communities was made *before* the HPTN 071 (PopART) trial results were known to the study team and was based on the expectation that the PopART intervention in arm A communities would have the greatest impact on ART coverage and HIV incidence.

#### Sampling for the TB infection cohort

For the sampling frame we used enumeration data collected in 2013 from the HPTN 071 (PopART) trial which provided the location of each household in the community. Irrespective of the size of the community, the area was subdivided into 10 equally sized zones. Every zone was further subdivided into blocks of approximately 40 households in Zambia and 55 in SA, on the basis that this was expected to result in an average of ~40 individuals aged 15–24 years enrolled in each randomly sampled block in both countries. To ensure geographic representativeness a two-stage sampling process was applied: (1) the 10 zones were put in a random order and (2) one block of households was randomly selected from each zone. The ten selected blocks were visited sequentially. All households within a selected block were eligible and approached until the target of 300 consenting AYA per community was reached. In case the target was not met after visiting the first ten blocks of households, new blocks were randomly selected from the same zones by repeating the second stage of the sampling described above.

### Study procedures

#### Enrolment

Enrolment took place from July- December 2018 in Zambia and from November 2018- July 2019 in SA. Permission for enumeration was sought from the responsible adult. Household members between 15–24 years of age who had lived in the community for 2 years or more and were expecting/planning to be resident for at least 9 months per calendar year for the next 2 years, were eligible to participate. Eligible individuals were invited to be seen at a clinical research site at either the clinic (Zambia) or in the community (SA).

#### Baseline assessment

At enrolment participants were interviewed by a research assistant using a structured questionnaire that included demographic, socioeconomic, and behavioural data as well as data related to HIV prevention, diagnosis, and treatment. Physical measurements of height and weight were taken.

A TB symptom screen (continuous cough >2 weeks, fever > 1 week, unintentional weight loss, drenching night sweats, chest pain, haemoptysis, shortness of breath) was administered. Participants were educated on the signs and symptoms of TB and asked to report to the local health facility if they developed any TB symptoms. Symptomatic participants provided sputum samples for Xpert MTB/RIF Ultra (Cepheid, Sunnyvale, USA) testing. Participants who were Xpert positive were referred to the local clinic for further management. Blood was drawn for QuantiFERON-TB Gold Plus (QFT-Plus; Qiagen, Hilden, Germany) testing and rapid HIV testing [21]. In Zambia, laboratory HIV testing was additionally conducted for those who did not self-report HIV status and who declined on-the-spot rapid test.

#### QuantiFERON-plus testing

QFT-Plus testing was performed according to the manufacturer’s instructions [21]. Blood was collected for QFT-Plus testing in lithium heparin tubes stored at room temperature. Blood was aliquoted into the four QFT-plus tubes and incubated for 16–24 hours at 37°C after which plasma was harvested and frozen at –20°C. Samples were processed within 24-hours. In 6/8 communities in Zambia the incubation step was conducted at regional laboratories. These samples were then transferred to a central research laboratory in Lusaka monthly for the enzyme linked immunosorbent assay (ELISA) performed on batched samples. In the remaining 2/8 of the Zambian communities both incubation and ELISA steps were carried out at the central research laboratory in Lusaka. In SA heparinized samples from all communities were transported to a central laboratory in Cape Town for incubation and then to Johannesburg for ELISA. In both countries the QFT-Plus Analysis Software was used to calculate results.

#### Follow up

All study procedures were repeated at 12 and 24 months follow up visits. Xpert testing was offered for participants symptomatic for TB at 12 months and for all participants irrespective of symptoms at 24 months. To reduce losses to follow-up in the cohort, we collected locator information (home address; phone numbers of participants and next of kin) during each visit. Participants were encouraged to attend their appointments through phone call reminders. Those who missed their appointments were followed up at home. Participants who moved out of their community during the study period were traced and encouraged to attend follow up.

We also conducted mid-year phone calls at which participants were given the blood test results from the previous visit and a TB symptom screen was administered. TB infection results were reported back to participants at 6, 18 and 25 months. Participants who tested HIV positive and QFT-Plus positive according to manufacturer’s cut-off values, were offered TB preventive therapy (TPT) and linked to HIV care.

### Statistical analysis

#### Definition of the outcome

The predefined primary outcome was the incidence of TB infection comparing arm A with arm C communities measured in the cohort of approximately 4200 AYA aged 15–24 years.

To account for within-person and between-test variation, and informed by prior analysis of longitudinal data among household contacts of TB cases in the ZAMSTAR trial [22], an incident case of TB infection was defined as a participant with QFT interferon-gamma test result of < 0.2 IU/ml plasma (‘negative’) at baseline and a QFT interferon-gamma (IFN-g) response of ≥0.7 IU/ml (‘positive’) in the 12 or 24 months follow up sample. The IFN-gamma concentrations of both TB1 and TB2 tubes needed to be < 0.2 IU/ml plasma to be classified as ‘negative at baseline’ and a sample was classified as ‘positive at 12 or 24 months’ if at least one of the TB1 or TB2-tubes had an IFN-gamma concentration of ≥0.7 IU/ml plasma (*double* the manufacturers cut point-off point of 0.35 IU/ml). For the classification of QFT-negative and QFT-positive, all IFN-gamma measurements were adjusted for the negative control.

The *M*. *tuberculosis* (*Mtb)* infection incidence rate was defined as the number of incident cases of infection divided by the person-years of follow-up, restricted to those with a QFT-Plus negative result at baseline and at least one valid QFT-Plus test at 12 or 24 months. For incident cases of infection, the timing of the infection was placed at the mid-point between last QFT-Plus negative test result and first positive QFT-Plus result. For individuals without incident infection their follow up time was censored at the last valid negative QFT-Plus test result.

#### Sample size

Study power was calculated using standard formulae for pair-matched cluster-randomized trials. With a sample size of 4,200 AYA across 14 communities (300 per community) and assuming 60% were negative for *Mtb* infection at baseline, 180 AYA per community would be followed up to measure the incidence of infection. If 90% were followed up at 12 months and 80% at 24 months, this would provide approximately 300 person-years of follow up time per community. With a coefficient of between-community variation (k) of 0.15, and an *Mtb* infection incidence rate of 5–6 infections per 100 person-years in standard-of-care communities, study power was 92% to show a reduction in incidence rate of 50% in the intervention communities. Likewise, study power was 86% to detect a reduction in incidence rate of 50% in the intervention communities if k was 0.20.

#### Intervention effect

The comparison between Arm A and Arm C was made by estimating the rate ratio (RR) comparing Arm A with Arm C. In our analysis we used standard analysis techniques for cluster-randomized trials with <15 clusters per trial arm [23]. We calculated the rate-ratio of TB-infection using the geometric mean of the community-level rates of TB-infection of arm A communities in the numerator and arm C communities in the denominator.

In unadjusted analysis we fitted a linear regression model of the log community-level rate on triplet and trial arm, to obtain a rate ratio comparing Arm A with Arm C that accounts for the matched trial design. The standard error of the rate ratio comparing Arm A with Arm C was calculated using the residual mean square from the regression model, with 6 degrees of freedom. The *t*-distribution was used to obtain *p*-values and 95% confidence intervals for the rate ratio.

The adjusted analysis was done using a 2-stage process. A Poisson regression model was fitted to the individual-level data (stage 1) for Zambia and SA data separately. Explanatory variables included in this regression model were triplet (to respect the trial design), sex and age group (15–19 years, 20–24 years). The fitted regression model was used to obtain the expected number of new infections with *Mtb* in each community under the null hypothesis of no intervention effect. For cluster level analysis (stage 2), for each community we calculated the ratio of observed (O) to expected (E) infections (O/E), and then calculated the log(ratio-residual) as log(O/E). We fitted a linear regression model of log(O/E) on triplet and trial arm, to obtain a rate ratio comparing Arm A with Arm C. In age and sex subgroups analysis, the above adjusted analysis was repeated without adjusting for sex and age group respectively at stage 1 of the analysis.

Sensitivity analysis was conducted using alternative definitions of incident infection with *Mtb* based on QFT results at baseline and follow-up, as informed by other studies [24, 25], and by including individual HIV status, community-level prevalence of *Mtb* infection and education in the Poisson regression model at stage 1 of the adjusted analysis.

### Ethical considerations

Participants aged ≥18 years provided written consent. Individuals <18 years provided written assent, with written consent provided by their parent or guardian, before participating in the study. Ethical approval for the trial was granted by ethics committees at the London School of Hygiene & Tropical Medicine (LSHTM), University of Zambia (UNZABREC) and the Pharma-Ethics Committee in Pretoria, SA.

## Results

### Enrolment and follow-up

**Fig 1:**
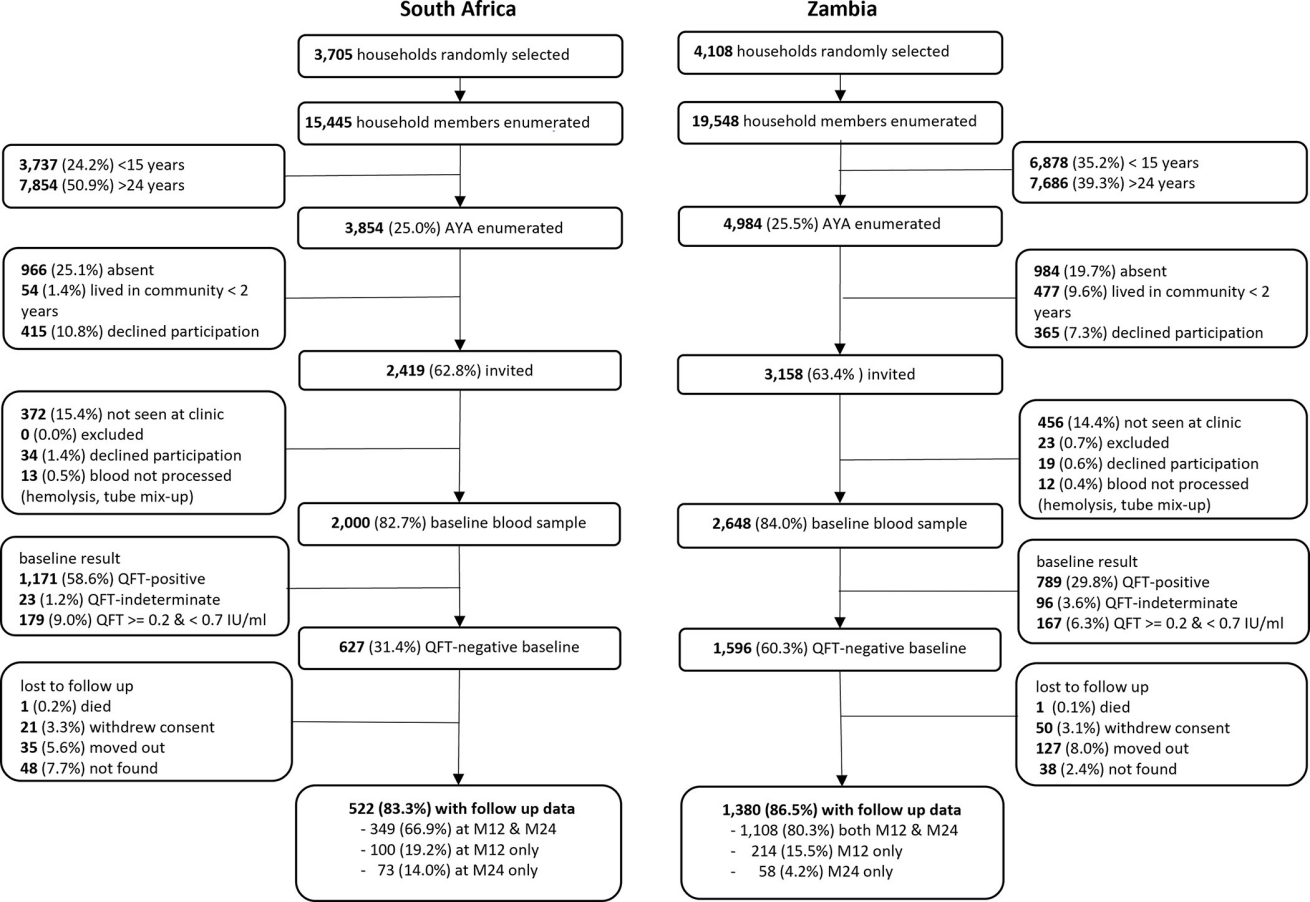
Enrolment and follow-up of the infection cohort.

Across all 14 communities, we randomly sampled 7,813 households in which 34,993 household members were enumerated (15,445 in SA and 19,548 in Zambia). In the 6 SA communities, 3,854 AYA matched the age criteria for participation, 2,419 were invited and 2,000 were consented and provided a baseline blood sample. In the 8 Zambia communities, 4,984 AYA matched the age criteria for participation, 3,158 were invited and 2,648 consented and provided a baseline blood sample *(*Fig 1).

In SA of the 2,000 AYA consenting at baseline, 627 (31.4%) had a negative QFT-plus result and 522/627 (83.3%) had at least one blood sample at follow up visits. Most of the AYA (349/522, 66.9%) had a blood sample at both 12 and 24 month follow up, 19.2% (100/522) a blood sample at 12-months only and 14.0% (73/522) at 24-months only. In Zambia of the 2,648 AYA consenting at baseline, 1,596 (60.3%) had a negative QFT-plus result and 1,380/1,596 (86.5%) had at least one blood sample at follow up visits. Most of the AYA (1,108/1,380 80.3%) had a blood sample at both 12 and 24 month follow up, 15.5% (214/1,380) a blood sample at 12-months only and 4.2% (58/1,380) at 24-months only.

### QuantiFERON results at baseline

Among all participants from arm A communities (combined) that had a follow-up QFT-results available, 42.6% tested QFT-positive at baseline compared to 42.3% in individuals from arm C communities (Table 1). The proportion QFT-positive in Zambia was approximately half of the proportion QFT-positive in SA (30.6% arm A, 28.6% arm C in Zambia versus 59.4% arm A, 59.9% in arm C in SA).

**Table 1:** QuantiFERON-result at baseline by arm and country for participants with follow-up QuantiFERON-result available.

	Arm A		Arm C	
	N	%	N	%
**All**	1996	*100*.*0%*	1980	*100*.*0%*
• *QFT-positive*	850	*42*.*6%*	837	*42*.*3%*
• *QFT-negative*	947	*47*.*4%*	955	*48*.*2%*
• *QFT >= 0*.*2 and < 0*.*7 IU/ml*	150	*7*.*5%*	137	*6*.*9%*
• *Indeterminate*	49	*2*.*5%*	51	*2*.*6%*
**Zambia**	1162	*100*.*0%*	1113	*100*.*0%*
• *QFT-positive*	355	*30*.*6%*	318	*28*.*6%*
• *QFT-negative*	695	*59*.*8%*	685	*61*.*5%*
• *QFT >= 0*.*2 and < 0*.*7 IU/ml*	73	*6*.*3%*	68	*6*.*1%*
• *Indeterminate*	39	*3*.*4%*	42	*3*.*8%*
**SA**	834	*100*.*0%*	867	*100*.*0%*
• *QFT-positive*	495	*59*.*4%*	519	*59*.*9%*
• *QFT-negative*	252	*30*.*2%*	270	*31*.*1%*
• *QFT >= 0*.*2 and < 0*.*7 IU/ml*	77	*9*.*2%*	69	*8*.*0%*
• *Indeterminate*	10	*1*.*2%*	9	*1*.*0%*

### Balance across arms

Among QFT-negative participants some differences were observed between the arms in socio-demographic factors and HIV-status (Table 2): arm A had a lower proportion of male participants (41.6% male in arm A versus 45.9% arm C), a higher proportion of younger ages (29.8% aged 15–16 years in arm A versus 25.1% in arm C), a higher proportion of HIV-negative participants (88.3% in arm A versus 80.3% in arm C) and a lower proportion of AYA with completed secondary education (73.1% in arm A versus 88.5% in arm C).

**Table 2:** Balance of socio-demographic characteristics and HIV-status across the arms among those QFT-negative at baseline with follow up QuantiFERON-result available.

	Arm A		Arm C	
	N	%	N	%
**All**	947	*100*.*0%*	955	*100*.*0%*
**Sex**				
*Male*	394	*41*.*6%*	438	*45*.*9%*
*Female*	553	*58*.*4%*	517	*54*.*1%*
**Age**				
*15–16*	282	*29*.*8%*	240	*25*.*1%*
*17–18*	223	*23*.*5%*	222	*23*.*2%*
*19–20*	183	*19*.*3%*	199	*20*.*8%*
*21–22*	142	*15*.*0%*	160	*16*.*8%*
*23–24*	117	*12*.*4%*	134	*14*.*0%*
**HIV-Status**				
*Tested negative*	836	*88*.*3%*	767	*80*.*3%*
*Self-Reported HIV positive*	12	*1*.*3%*	13	*1*.*4%*
*Tested HIV positive*	21	*2*.*2%*	32	*3*.*4%*
*Unknown*	78	*8*.*2%*	143	*15*.*0%*
**Education** *^)^				
*Less than primary*	44	*4*.*7%*	7	*0*.*7%*
*Primary*	209	*22*.*2%*	103	*10*.*8%*
*Secondary*	688	*73*.*1%*	843	*88*.*5%*
**HH size**				
*1*	7	*0*.*7%*	4	*0*.*4%*
*2*	52	*5*.*5%*	60	*6*.*3%*
*3*	107	*11*.*3%*	93	*9*.*8%*
*4*	136	*14*.*4%*	129	*13*.*6%*
*5 or more*	645	*68*.*1%*	669	*70*.*6%*

*^)^ 6 missing for Arm A, 2 missing for Arm C

### TB infection incidence rates and effect of the PopART intervention

Among the 1,902 QFT-negative participants at baseline, 213 converted from QFT-negative at baseline to QFT-positive at 12 or 24 months. With a total follow up time of 2,987 person-years, this gives an overall crude TB-incidence infection rate of 7.1 per 100 person-years (Table 3). The crude TB-infection rate was much higher in SA (17.3 per 100 person-years) than in Zambia (4.2 per 100 person-years), higher in male AYA (7.9 per 100 person-years) than in female AYA (6.5 per 100 person-years) and was similar across age groups (7.0 and 7.3 per 100 person-years for 15–19 and 20-24-year-olds respectively).

**Table 3:** TB-infection rates by community, country, sex and age groups: Overall and stratified by trial arm.

	Incident cases ^1)^ / Total person-years *(rate per 100 person-years)*		
	Arm A and C combined	Arm A	Arm C
Triplet 1	22/685 *(3*.*2)*	15/329 *(4*.*6)*	7/356 *(2*.*0)*
Triplet 2	30/563 (5.3)	18/289 *(6*.*2)*	12/275 *(4*.*4)*
Triplet 3	34/420 *(8*.*1)*	21/228 *(9*.*2)*	13/192 *(6*.*8)*
Triplet 4	12/654 *(1*.*8)*	5/322 *(1*.*6)*	7/332 *(2*.*1)*
Triplet 5	45/229 *(19*.*7)*	18/84 *(21*.*3)*	27/144 *(18*.*7)*
Triplet 6	32/238 *(13*.*5)*	23/116 *(19*.*8)*	9/122 *(7*.*4)*
Triplet 7	38/197 *(19*.*3)*	24/113 *(21*.*2)*	14/84 *(16*.*7)*
Overall	213/2,987 *(7*.*1)*	124/1,482 *(8*.*4)*	89/1,505 *(5*.*9)*
SA	115/664 *(17*.*3)*	65/314 *(20*.*7)*	50/350 *(14*.*3)*
Zambia	98/2,323 *(4*.*2)*	59/1,168 *(5*.*1)*	39/1,155 *(3*.*4)*
Male AYA	104/1,317 *(7*.*9)*	56/622 *(9*.*0)*	48/696 *(6*.*9)*
Female AYA	109/1,669 *(6*.*5)*	68/860 *(7*.*9)*	41/809 *(5*.*8)*
15–19 years	128/1,820 *(7*.*0)*	78/950 *(8*.*2)*	50/870 *(5*.*8)*
20–24 years	85/1,167 *(7*.*3)*	46/531 *(8*.*7)*	39/636 *(6*.*1)*

^1)^ Incident case is defined as converting from QFT-negative at baseline (< 0.2 IU/ml) to QFT-positive at 12 or 24 month follow up (> = 0.7 IU/ml)

**Fig 2:**
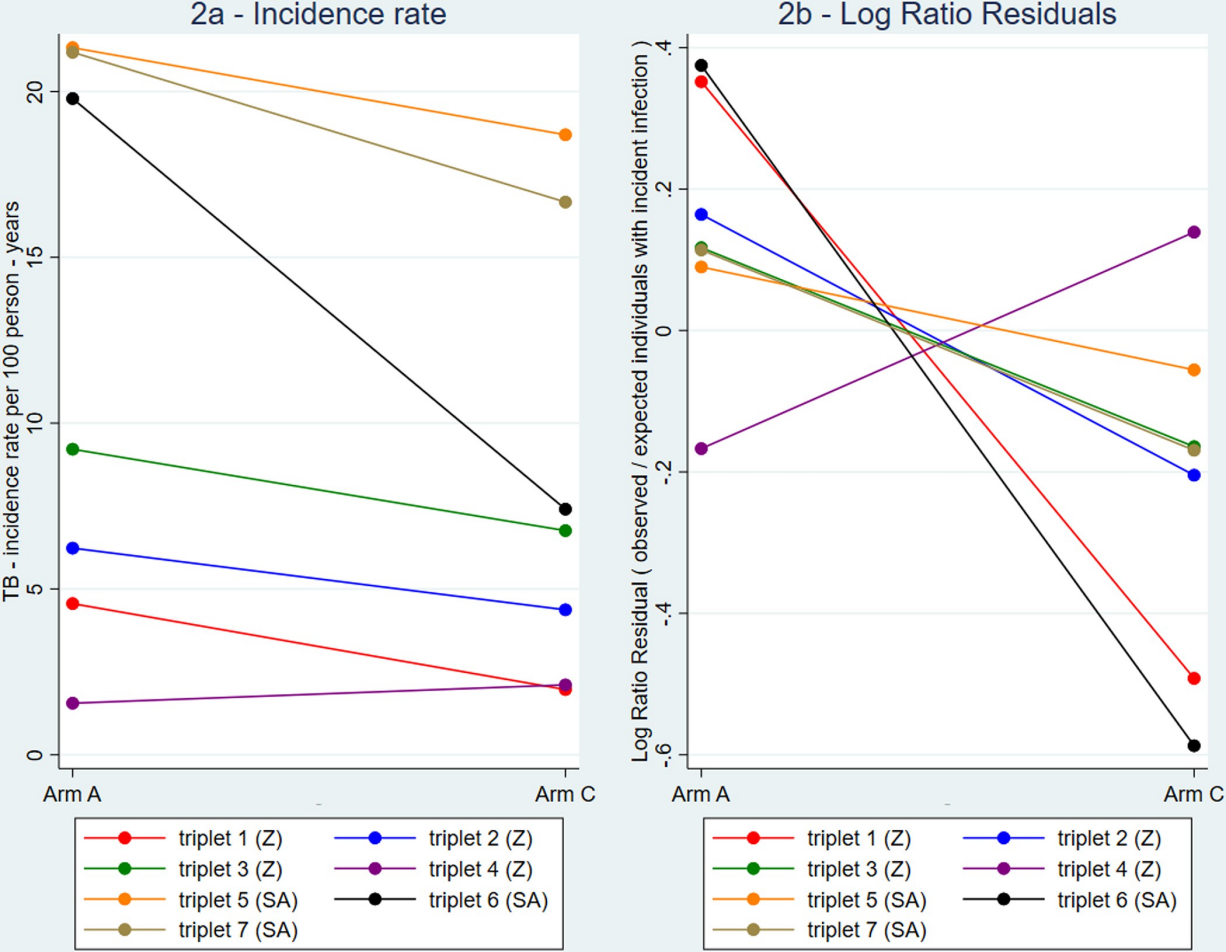
Incidence rates of TB infection per 100 person-years (2a) and estimates of the Log Ratio Residuals (2b) comparing the arm A and arm C community in each of the seven triplets.

Considering participants by arm, the individuals in arm A communities had a higher crude TB-infection incidence rate (8.4 per 100 person-years) compared to arm C communities (5.9 per 100 person-years). Fig 2A shows that for every triplet except triplet 4, the Arm A community had a *higher* crude TB-infection incidence rate than the corresponding arm C community.

For our primary definition of the outcome of incident *Mtb* infection, the incidence in arm C (geometric mean across communities) was 6.0 per 100 person-years (Table 4) and in arm A was 8.7 per 100 person-years; giving an adjusted rate ratio (aRR) of 1.45 (95% confidence interval [CI], 0.97–2.15; P = 0.063). In adjusted analysis, Fig 2B shows that for every triplet except triplet 4, the log ratio residuals were higher in the arm A community than the corresponding arm C community.

**Table 4:** Rate ratio of incidence of TB-infection comparing arm A with arm C communities.

	TB-infection rate per 100 person-years*^)^		Unadjusted			Adjusted for age and sex		
	Arm A	Arm C	Rate Ratio	95% CI	p-value	Rate Ratio	95% CI	p-value
Overall	8.7	6.0	**1.44**	**0.96–2.15**	**0.068**	**1.45**	**0.97–2.15**	**0.063**
SA	20.8	13.2	1.57	0.74–3.34	0.123	1.59	0.78–3.24	0.107
Zambia	4.5	3.3	1.35	0.78–2.37	0.189	1.35	0.76–2.37	0.195
Male	8.7	7.0	1.25	0.50–3.11	0.576	1.24 ^1)^	0.51–3.04	0.576
Female	8.1	4.8	1.70	1.29–2.25	0.003	1.67 ^1)^	1.28–2.17	0.003
Age 15–19	8.8	5.8	1.51	1.01–2.27	0.047	1.53 ^2)^	1.03–2.27	0.038
Age 20–24	8.2	6.2	1.33	0.68–2.59	0.337	1.35 ^2)^	0.67–2.73	0.338

*^)^ Geometric mean of the cluster rates

^1)^ Adjusted for age only

^2)^ Adjusted for sex only

In subgroup analysis the adjusted rate ratio (aRR) was higher in SA (aRR = 1.59, 95%CI 0.78–3.24, p = 0.107) than Zambia (aRR = 1.35, 0.76–2.37, p = 0.195). There was some evidence that in intervention communities the incidence of TB infection was higher than in standard-of-care communities among female AYA (aRR = 1.67, 95%CI 1.28–2.17, p = 0.003). There was no evidence of a difference between intervention and standard-of-care communities in male AYA (aRR 1.24, 95%CI 0.51–3.04, p = 0.576). However, the observed difference in intervention effect between males and females could be due to chance (test for interaction, p = 0.407). Similarly, there was weak evidence that incidence of TB-infection was higher in intervention compared with standard-of-care communities among individuals aged 15–19 years (aRR = 1.53, 95% CI 1.03–2.27, p = 0.038) but there was no evidence of a difference among older individuals aged 20–24 years (aRR = 1.35, 95%CI 0.67–2.73, p = 0.338 test for interaction p = 0.640).

### Sensitivity analysis

**Fig 3:**
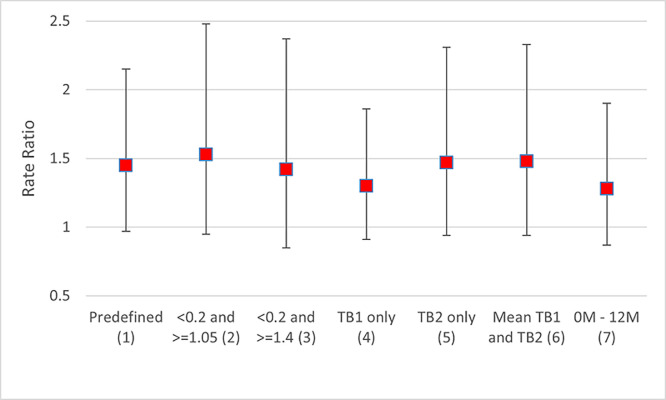
Adjusted rate ratio of incidence of TB-infection comparing arm A with arm C communities using alternative definitions of incident TB-infection incident (sensitivity analysis). (1) Predefined analysis with incident case of TB-infection defined as a participant with a baseline QFT IFN-gamma response <0.2 IU/ml for both TB1Nil and TB2Nil tube and an IFN-gamma response of > = 0.7 IU/ml in either TB1Nil or TB2Nil in a subsequent blood sample at 12- or 24-month follow-up. (2) Same as (1) but using 1.05 IU/ml instead of 0.7 IU/ml. (3) Same as (1) but using 1.4 IU/ml instead of 0.7 IU/ml. (4) Same as (1) but only considering the TB1Nil IFN-gamma response. (5) Same as (1) but only considering the TB2Nil IFN-gamma response. (6) Same as (1) but using the mean of the IFN-gamma responses of TB1Nil and TB2Nil. (7) Analysis restricted to TB-incidence between 0 and 12 months using same definition of TB-incident case as in (1).

**Fig 4:**
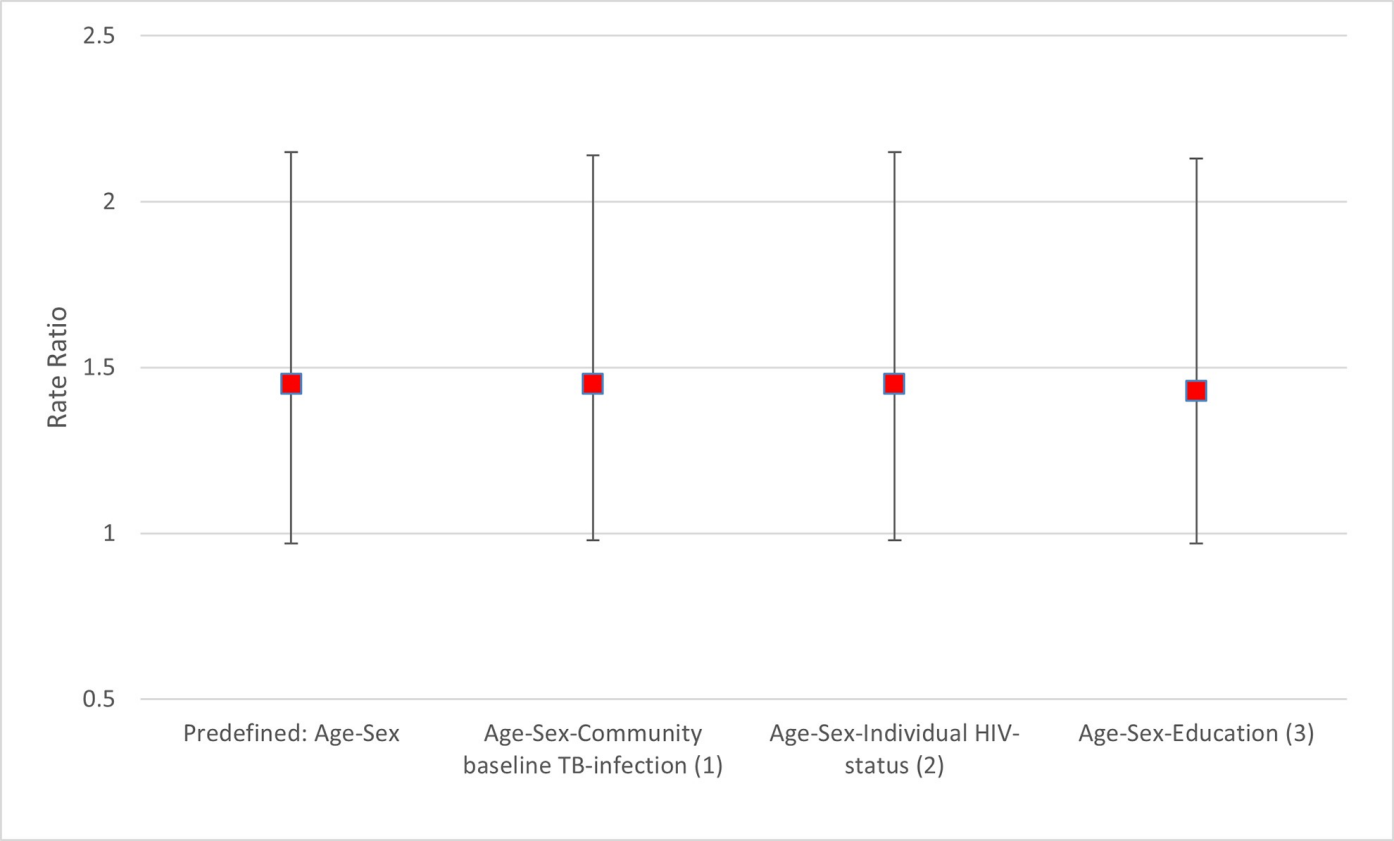
Rate ratio of incidence of TB-infection comparing arm A with arm C communities expanding the number of characteristics to adjust for at stage one of the two-stage cluster level analysis (sensitivity analysis). (1) TB-infection defined as those with an IFN-gamma response of > 0.7 IU/ml at baseline among all with at least one follow up sample at 12- and/or 24 months. (2) Individual HIV-status at baseline as reported in Table 2. (3) Education as reported in Table 2.

Fig 3 shows the aRR of the incidence of TB-infection comparing intervention and standard-of-care communities using alternative definitions based on QFT results at baseline and during follow-up. Fig 4 show the aRRs adjusted for baseline community-level prevalence of QFT-positivity, individual HIV-status and education. The aRRs for each of these sensitivity analyses were similar to the aRR of the primary analysis.

## Discussion

TREATS was designed to measure whether a universal testing and treatment intervention for HIV, with population-level screening for TB, could reduce TB prevalence, TB incidence and TB transmission in urban, high prevalence communities. Our community-randomized study found no evidence that a combined TB/HIV prevention intervention reduced the incidence of TB infection among AYA at population level. Several factors could have contributed to the apparent lack of impact of the PopART intervention on TB infection incidence.

The TB specific component of the PopART intervention consisted of systematic TB screening based on TB symptoms. This strategy may not have been sensitive enough to reach a high screening efficiency and reduce TB transmission at community level. Modelling exercises have suggested that a reduction of TB infection incidence can be achieved with a screening efficiency (proportion of undiagnosed TB cases identified through the TB screening strategy) of 30% or more (S1 Table). Our sample size calculations were based on minimally 50% reduction of TB infection incidence in intervention communities. In the ZAMSTAR trial, carried out from 2005 to 2011 in 24 communities in Zambia and the Western Cape of SA, a household intervention that included TB screening showed weak evidence of an effect [22]. However, it is well recognised that symptom screening alone may miss a large proportion of individuals with active prevalent TB because most individuals found with TB in prevalence surveys in high TB/HIV prevalent settings do not report TB symptoms [26–29]. The wider application of more sensitive TB screening algorithms, including the use of chest x-ray, at population level remains limited due to logistics and high operational costs.

The impact of the PopART intervention on TB, was also evaluated through the TREATS TB prevalence survey. With a prevalence ratio of 1.14 (95% confidence interval [0.67,1.95], p = 0.60) comparing intervention communities with control communities, there was no evidence of a reduction in TB prevalence [12, 30]. Since TB transmission is driven mostly by TB prevalence and the number (and susceptibility) of individuals with whom infectious individuals may come into contact, [31] it is thus not surprising that we found no evidence of an effect on the incidence of TB infection.

The PopART intervention improved ART coverage among HIV-positive individuals after four years of intervention, to approximately 81% in arm A communities [16]. Although ART reduces the incidence of TB (but not to the level of HIV-negative individuals), its effect on prevalence is less clear [32]. A growing proportion of PLHIV on ART with long survival times might make a considerable contribution to the TB burden at population level, as they may have a TB disease course that is more like that of HIV-negative individuals with higher infectiousness and longer duration of disease [33–35]. Modelling efforts will help to clarify these dynamics.

Little is known about the best timing to efficiently measure the impact on TB-infection rates after an intervention has been introduced. Modelling studies on active case finding suggest it might take several years before the impact is noticeable [36]. In our study, the cohort of AYA was enrolled approximately 18 months after the PopART intervention (that was delivered for four years) ended. However, it is possible that this timing of cohort enrolment and follow-up was not optimal. The COVID-19 pandemic also occurred during the follow-up period, the effect of which is difficult to assess.

Robust evidence is still lacking about how best to measure the incidence of TB-infection at population level. While repeated testing of uninfected individuals over time is a conventional method for determining incidence of a disease, this methodology is both labour and time intensive, and is further complicated in TB since only indirect measures based on the immune response to infection are available, and these may be affected by the boosting of the immune response, especially with in-vivo tests such as the TST [37, 38]. Traditionally, alternative approaches for calculating incidence from cross-sectional data have been used [39]. The annual risk of TB infection (ARTI) is based on a measure of the risk of TB infection averaged over the lifetime of the study participants, and so has traditionally used data from young children [39–41]. We chose to measure incidence of TB-infection in AYA to identify recent infections and compare the incidence of TB-infection across the arms. Based on previous knowledge of ARTI and contact patterns in Zambia and SA, we expected a substantial proportion of non-infected individuals among AYA [22, 39, 42]. However, we found a higher prevalence of TB-infection at baseline than had been anticipated, leaving us with a lower proportion of uninfected individuals (2,223 / 4,648, 47.8% across both countries) than assumed in sample size calculations (60%). A high proportion (71%) of QFT-positives were also found among AYA 12–17 year old with neonatal BCG-vaccination participating in a vaccine trial in SA [43]. Furthermore, AYA are thought to have increased ARTI compared to other age groups (32,33). More outside household activities are considered to be responsible for the increased contact patterns in AYA, assumed to be more representative for community TB-transmission than TB-infection in young children in whom household transmission may play a dominant role [13, 14].

We used the most recent test of TB infection, the QFT-Plus, as this does not have problems such as immune boosting and is reliable in PLHIV [44]. To our knowledge, we are the first to use QFT-Plus for the purpose of measuring the incidence of TB infection at population level. Although IGRAs offer an alternative method of serial testing to detect new TB infection, they are generally used in clinical settings [14, 25].

IGRAs have a wide-range of sources of variability that can have an impact on the result such as blood volume, laboratory and transportation related issues [45]. In addition, there is limited evidence regarding the reproducibility of QFT-Plus in LMICs [46, 47]. Although we applied stringent quality assurance and quality control measures, we cannot entirely rule out variability due to different laboratories and processing. To avoid challenges from test variability we used a stringent QFT-Plus conversion definition due to possible discordance on serial testing [45]. We followed recent suggestions in the literature to avoid uncertainty around the cut-point by having our endpoint use conversion from <0.2 to > = 0.7 [24, 48–50]. To date only two large studies have provided evidence which supports the use of a stringent QFT conversion definition excluding the proposed zone of uncertainty [24, 25]. Our sensitivity analysis showed that using alternative IFN-g cut-off values to define an incident TB infection gave similar estimates of intervention effect.

We saw slight differences between the trial arms in HIV status, age and education. Accounting for these differences in adjusted and sensitivity analysis, did not change our main findings. As postulated in the overall PopART trial findings [11], although the three trial arms appeared well matched at baseline, there may have been unrecognized differences across communities in sociodemographic or other factors, such as mobility and migration. Although these urban communities had high mobility, analysis of available data did not suggest any appreciable differences in migration across the trial groups [11]. Furthermore, the HPTN 071 (PopART) communities were matched on HIV-prevalence. Randomization was further restricted to ensure balance across the study arms on cluster size and ART uptake. Direct baseline TB measures were not part of the randomization process. Although baseline QFT-positivity was well balanced across the study arms, we cannot rule out community differences in pre-intervention TB transmission rates and thus—with a relatively small number of clusters–the study could have been susceptible to random error.

## Conclusion

This community-randomized study found no evidence that a combined TB/HIV prevention intervention reduced the incidence of TB infection at population level. In our trial setting, we did not observe a reduction in TB transmission after UTT combined with active case finding for TB was implemented. Our data will inform future modelling work to better understand the population level dynamics of HIV and TB.

## Supporting information

S1 Fig Overview of study design and relationship to HPTN 071. (TIF)

S1 Table The modelled effect of the HPTN071 (PopART) interventions on TB disease prevalence and the incidence of TB infection. (DOCX)
