# Supplementary figures and images for: The impact of a combined TB/HIV intervention on the incidence of TB infection among adolescents and young adults in the HPTN 071 (PopART) trial communities in Zambia and South Africa
Source: PLOS Glob Public Health. 2023 Jul 14;3(7):e0001473. doi: 10.1371/journal.pgph.0001473 (PMC10348566; doi:10.1371/journal.pgph.0001473)

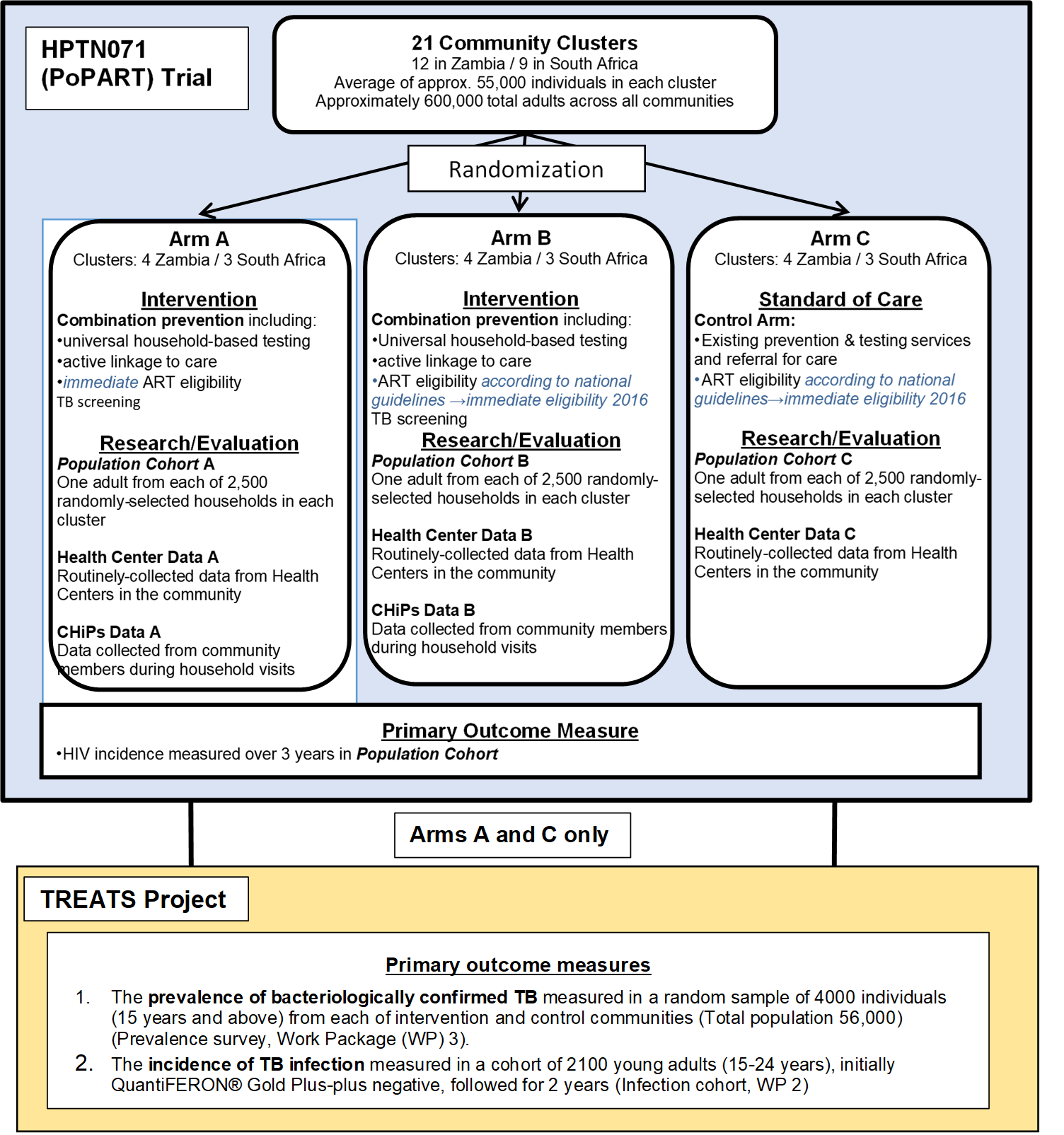

Supplement: S1 Fig — (TIF) [file pgph.0001473.s001.tif]
