# Supplementary material for: The impact of a combined TB/HIV intervention on the incidence of TB infection among adolescents and young adults in the HPTN 071 (PopART) trial communities in Zambia and South Africa
Source: PLOS Glob Public Health. 2023 Jul 14;3(7):e0001473. doi: 10.1371/journal.pgph.0001473 (PMC10348566; doi:10.1371/journal.pgph.0001473)
Supplement: S1 Table — (DOCX) [file pgph.0001473.s002.docx]

**S1 Table:** **The modelled effect of the HPTN071 (PopART) interventions on TB disease prevalence and the incidence of TB infection**

Using mathematical modelling the likely effect of the intervention on the co-primary outcomes of the TREATS study [1], which are the prevalence of active TB among individuals aged ≥15 years and the incidence of new infection with *M. tuberculosis* among young adults aged 15-24 years, was estimated.

Depending on the efficiency of active case finding, a 30%-60% reduction in both adult TB prevalence and the incidence of new infection with *M. tuberculosis* among young adults was projected.

| Screening efficiency | HIV diagnosis/linkage | Effect on disease prevalence (risk ratio)* | Effect on incidence of infection (risk ratio)^§^ |
| --- | --- | --- | --- |
|  |  | Arm A/C | Arm A/C |
| 10 | 40 | 0.68 | 0.71 |
| 20 | 40 | 0.59 | 0.60 |
| 30 | 40 | 0.51 | 0.51 |
| 40 | 40 | 0.44 | 0.44 |
| 10 | 50 | 0.66 | 0.69 |
| 20 | 50 | 0.57 | 0.59 |
| 30 | 50 | 0.49 | 0.50 |
| 40 | 50 | 0.43 | 0.43 |
| 10 | 60 | 0.64 | 0.68 |
| 20 | 60 | 0.55 | 0.58 |
| 30 | 60 | 0.48 | 0.49 |
| 40 | 60 | 0.42 | 0.42 |

*Changes in disease prevalence modelled in the intervention (A) and control (C) arms over 73 months from a baseline prevalence of 832/100,000 population (a minimum TB disease prevalence estimate for the communities from the ZAMSTAR trial), and prevalence at month 63 in the intervention (A) and control (C) arms are compared; the TB active case-finding rounds of the of the HPTN071 (PopART) intervention were modelled at 8, 24 and 40 months. ^§^Comparison between the cumulative incidence of TB infection modelled in the intervention (A) and control (C) arms over the 24 months between month 48.5 and month 72.5, used to capture the duration of the incidence of TB infection cohort. screening efficiency=proportion of undiagnosed TB cases identified through the TB screening strategy; HIV diagnosis/linkage=proportion of PLWH not on ART who are identified and started on ART; ACF=active case finding; PLWH= people living with HIV; ART=antiretroviral therapy

**Reference**

1. European and Developing Countries Clinical Trials Partnership (EDCTP). TREATS Study funded by EDCTP, RIA 2016S-1632 2018 [Available from: <https://publications.edctp.org/international-partnerships-against-infectious-diseases/treats>.
